# A comparative view of regenerative neurogenesis in vertebrates

**DOI:** 10.1242/dev.122796

**Published:** 2016-03-01

**Authors:** Alessandro Alunni, Laure Bally-Cuif

**Affiliations:** Paris-Saclay Institute for Neuroscience, CNRS UMR9197 – Université Paris-Sud, Université Paris-Saclay, Team Zebrafish Neurogenetics, Avenue de la Terrasse, Building 5, Gif-sur-Yvette F-91198, France

**Keywords:** Lesion, Neural stem cells, Neurogenesis, Repair, Zebrafish

## Abstract

In all vertebrate species studied thus far, the adult central nervous system harbors neural stem cells that sustain constitutive neurogenesis, as well as latent neural progenitors that can be awakened in lesional contexts. In spite of this common theme, many species differ dramatically in their ability to recruit constitutive progenitors, to awaken latent progenitors, or to enhance or bias neural progenitor fate to achieve successful neuronal repair. This Review summarizes the striking similarities in the essential molecular and cellular properties of adult neural stem cells between different vertebrate species, both under physiological and reparative conditions. It also emphasizes the differences in the reparative process across evolution and how the study of non-mammalian models can provide insights into both basic neural stem cell properties and stimulatory cues shared between vertebrates, and subsequent neurogenic events, which are abortive under reparative conditions in mammals.

## Abstract (precis)

**Summary:** This Review article provides a comparative view of neuronal repair across vertebrate species, with a particular focus on the molecular pathways that enable repair in some, but not all animals.

## Introduction

Regenerative neurogenesis is the process by which neuronal production can be re-established or enhanced in the nervous system to restore specific functions. Understanding how regenerative neurogenesis is achieved in different contexts is essential for efforts to repair lesions and reverse degenerative events in a therapeutic setting, or even to treat some mental disorders. In addition, understanding the mechanisms involved in successful neuronal replacement can provide a fundamental insight into cell plasticity, reprogramming and the encoding of stem cell fate in physiological conditions.

Achieving functional neuronal regeneration from constitutive neural progenitors and/or awakened latent neural progenitors requires tight control over a number of parameters. For example, it is crucial to regulate inflammation in the injured or degenerated tissue, to manage epigenetic mechanisms, and to reduce the risk of tumorigenic transformation. In addition, certain specific features of the nervous system make neural regeneration particularly challenging compared with that of other organs. There is a huge diversity of neuronal subtypes, which means that an extensive variety of differentiated cells must be generated, unlike some other organs where only a few functional cell types are required to ameliorate disease or injury. Another challenge is that there are multiple steps of neural differentiation, and each step represents a cell fate choice that must be tightly controlled. Spatial elements add an additional layer of complexity: regional differences in neuronal and glial subtypes, vascularization, microglial complement, ventricular access and tissue thickness are just a few of the variables that impact the type of regeneration required and its efficiency. Finally, different injury types in the nervous system can trigger different outcomes. For example, at least in rodents, stab wounds and ischemia endow reactive astrocytes with progenitor potential, whereas degenerative disease models do not.

**Fig. 1.**
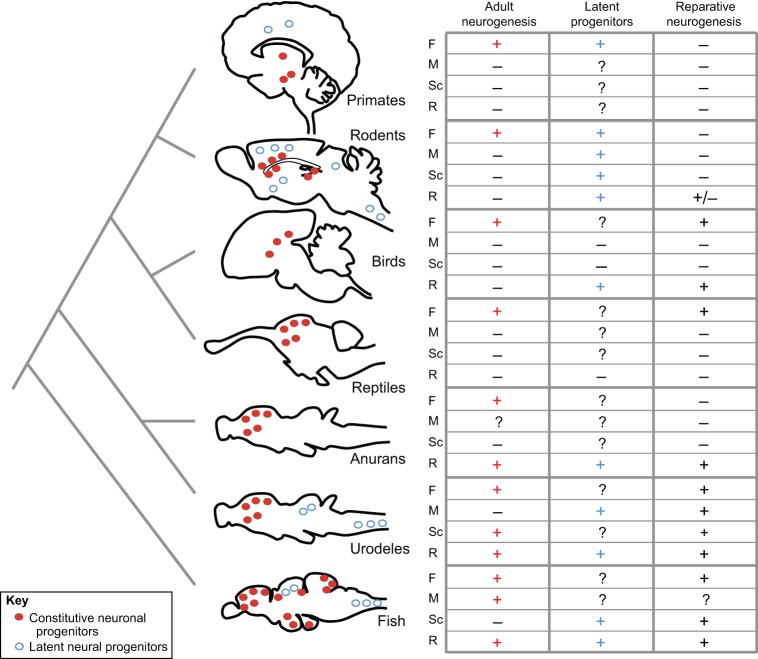
**Phylogenetic tree of animal taxons used as models for neuronal regeneration.** The location of adult neurogenic niches, which harbor constitutively active neuronal progenitors (red), and the presence of latent neural progenitors (blue) are indicated on schematic sagittal sections of the brain (left). Constitutive neurogenesis generates neurons in the adult brain under homeostatic conditions, whereas latent progenitors are activated in response to lesions to produce neurons and/or glial cells. The table summarizes the presence of (+), the demonstrated absence of (−), or the lack of experimental data on (?) constitutive neuronal progenitors, latent neural progenitors and reparative neurogenesis in the different central nervous system regions discussed throughout this Review. F, forebrain; M, midbrain; Sc, spinal cord; R, retina.

The many variables that affect mechanisms of repair in the human nervous system can be better understood by studying different vertebrate models, such as rodents (mouse or rats), birds, reptiles (lizards), urodele amphibians (axolotl and salamander) and teleost fish (principally zebrafish and medaka), that vary in the extent and efficiency of endogenous adult neurogenesis and their ability to regenerate (Fig. 1) (for a review, see Ferretti, 2011). Major differences exist between species with regard to the spatial domains where active neurogenesis occurs, as well as the response of latent progenitors upon injury or onset of disease. Overall, constitutively active neurogenic domains in rodents and birds are restricted to the forebrain and neurogenic niches therein, whereas they cover most of the forebrain ventricle in amphibians, and several brain subdivisions in teleost fish. Importantly, constitutively ‘silent’ areas also exist in all species, where progenitor potential can be revealed upon lesion, pathological conditions, transplantation or culture. Thus, the relevance and properties of both types of progenitors, constitutive and conditional, and their niches, neurogenesis-promoting and non-permissive, can be compared in an informative way between species.

In this Review, we focus on the production of new neuronal cells for neuronal regeneration, and discuss how comparisons between mammalian and non-mammalian neurogenesis and neuronal regeneration contribute to understanding regenerative neurogenesis. We first address the main mechanisms of endogenous neurogenesis and look at how this process can be hijacked to promote neuronal repair following injury both in non-mammalian and mammalian vertebrates. In further sections, we stress the similarities and differences between rodents and non-mammalian models in their ability to repair the nervous system from latent neural progenitors. To conclude, we discuss the stages of neural progenitor recruitment and neurogenesis that are either shared or divergent between species and consider the impact this has on regeneration, particularly for translation into humans.

We note that, in the context of reparative biology, the concepts of cell fate re-orientation and cell reprogramming, as well as the definition of a neural stem cell (NSC) or a differentiated cell can often become blurred. In most cases, the self-renewal properties of latent progenitors awakened during repair appear to be short-lived, or have not been assessed over time or upon repeated repair events. We will therefore use the more general term ‘progenitor cell’ instead of ‘stem cell’ when discussing these particular regeneration contexts. Another topic of caution, especially prominent in the neural regeneration field, is the generalization of ‘mouse’ to ‘mammals’ or, likewise, of ‘zebrafish’ to ‘fish’. At the risk of sounding restrictive, we will deliberately limit the conclusions drawn below to the very species from which they were obtained.

## Neuronal regeneration from constitutively active progenitors

Across species, the central nervous system harbors constitutively active neuronal progenitors that are responsible for producing new neurons throughout the life of the organism. These progenitors reside in what is known as active neurogenic zones (Fig. 1, red), which can be stimulated to contribute to neuronal repair. This has been observed in all vertebrate species studied thus far; however, differences between species are apparent with regard to the extent and efficiency of repair in different locations and injury contexts. Inter-species comparisons are an informative way to highlight common or divergent pathways that boost or limit neurogenic potential in various different settings.

### Constitutive neurogenesis from glial cells in the vertebrate nervous system

**Fig. 2.**
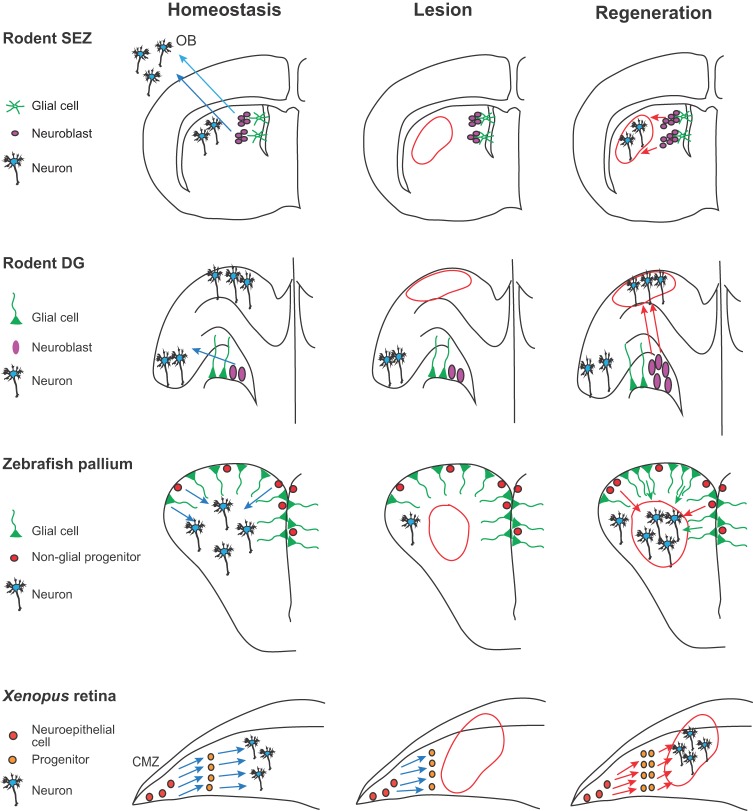
**Neuronal repair from niche progenitors.** In the rodent subependymal zone (SEZ), glial cells give rise to neuroblasts that migrate along the rostral migratory stream into the olfactory bulb (OB) to generate unique types of interneurons (left panel, blue arrows). Stroke injury (red outline) results in localized cell death in the striatum and the proliferation of endogenous neural progenitor cells that migrate from the SEZ to the striatum to elicit regeneration (right panel, red arrows). This migration occurs at the expense of normal neuroblast migration from the SEZ to OB. In the rodent dentate gyrus (DG), radial glial cells produce transit-amplifying progenitors, called neuroblasts, which generate neurons (left panel). These newborn neurons migrate into the granule cell layer (blue arrows). Ischemia (center panel, red outline) induces the degeneration of pyramidal neurons. Following the ischemia the endogenous progenitors proliferate and subsequently migrate to regenerate new neurons (right panel, red arrows). The ventricular zone of the adult zebrafish pallium consists predominantly of radial glial cells, which act as self-renewing and multipotent progenitors (left panel). In addition, non-glial cycling neuroblasts are intermingled along the ventricle. Together, radial glia and neuroblasts generate pallial neurons (left panel, blue arrows). Reactive neurogenesis has been induced in the zebrafish adult pallium mostly by mechanical injury using stab lesion causing a circumscribed injury in the parenchyma of the telencephalon without injuring ventricular lining (center panel, red outline). In response, neuroblasts and radial glia increase their proliferation to produce neurons to compensate for the neuronal loss (right panel, red and green arrows, respectively). In the amphibian retina, the ciliary marginal zone (CMZ) continuously generates all neuronal subtypes (left panel, blue arrows). Upon extensive lesion in *X. tropicalis* (center panel, red outline), the CMZ is activated to elicit regeneration (right panel, red arrows). In the rodent and zebrafish schematics, only the left hemisphere is depicted.

In mouse and rats, the two prominent niches of constitutive neurogenesis are the subependymal zone of the lateral ventricle (SEZ) and the subgranular zone of the dentate gyrus of the hippocampus (SGZ) (Fig. 1). The latter niche is the most active in humans (Bergmann et al., 2015), although a recent study identified new neurons that are constitutively added to the human striatum from a source yet to be defined (Ernst et al., 2014). Both the SEZ and SGZ contain a large heterogeneous population of endogenous active progenitor cells, which is dominated by astroglial cells, a type of non-neuronal cell important for homeostasis and support within neural tissues (recently reviewed by Bond et al., 2015; Kempermann et al., 2015). Although the precise hierarchical relationship operating within rodent germinal niches is still being teased apart (Bonaguidi et al., 2012), genetic-tracing studies support the idea that the glial cells are NSCs, and generate neurons via a series of intermediate, amplifying and non-glial cell states (Fig. 2). Compared with mammals, teleost fish such as zebrafish and medaka show significantly greater rates of neurogenesis, as new neurons are generated in most brain regions throughout adult life (reviewed by Kizil et al., 2012b; Schmidt et al., 2013). The dorsal telencephalon, otherwise known as the pallium, is an area of the teleost fish brain that contains regions homologous to the mammalian SEZ and SGZ as well as a neocortex-like region, where in rodents neural progenitors are silent. In the teleost pallium, neurogenic radial glial cells act as self-renewing and multipotent progenitors at the single-cell level, behaving as bona fide NSCs (Rothenaigner et al., 2011; reviewed by Than-Trong and Bally-Cuif, 2015). But not all cells are equal: a recent lineage-tracing study (Box 1) revealed differences in NSC densities and activation frequencies across the anterior, medial and lateral pallium under normal physiological conditions. This regional diversity in NSC activity is important to consider, especially when analyzing regeneration (Dray et al., 2015). In addition to NSCs, non-glial cycling neuroblasts, postulated equivalents of mammalian transit-amplifying progenitors, are intermingled along the zebrafish ventricle (März et al., 2010) (Fig. 2). It has been shown that the neurons generated from the pallial neurogenic zone populate the olfactory bulb and the pallium proper (Kroehne et al., 2011; Dirian et al., 2014); however, a more detailed spatiotemporal characterization of the heterogeneity of this neurogenic niche in terms of cell subtypes, lineages, division modes and fate has not yet been reported. In the adult pallium of both the red spotted newt (*Notophthalmus viridescens*) and the axolotl, ventricular radial glial cells also exhibit neurogenic potential and the capacity to retain a bromodeoxyuridine (BrdU) label – indicative of slow cell cycling – under physiological conditions (Berg et al., 2010; Maden et al., 2013). Radial glial heterogeneity has also been described in the newt (Kirkham et al., 2014), but the exact lineage relationship between these cell types and their relation to zebrafish or mammalian adult progenitors remains to be defined.

Box 1. A new perspective brought by non-mammalian models: live imaging of adult NSCs in their endogenous niche

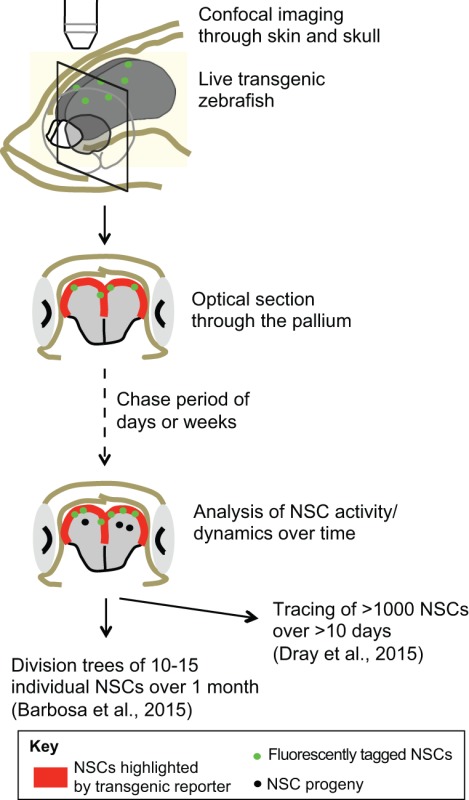

The zebrafish model allows dynamic imaging of adult NSCs in their endogenous niche using completely non-invasive methods (Barbosa et al., 2015; Dray et al., 2015). Transgenic fish devoid of pigments (White et al., 2008) can be crossed with transgenic lines that harbor fluorescently tagged NSCs (Yeo et al., 2007) combined with cell division markers or with transiently electroporated fluorescent tracers to track cell dynamics over time. This approach enables direct access to the ventricular surface of the zebrafish pallium, and has been used to study NSC dynamics both during normal physiological conditions (Dray et al., 2015) and during neuronal repair (Barbosa et al., 2015).

NSCs in all species are relatively quiescent compared with most other dividing cells: a mechanism that helps to protect NSCs from exhaustion. However, recruiting endogenous NSCs for repair will, in part, necessitate an exit from quiescence, and thus the regulation of quiescence is a topic of great interest for neural repair. Molecular analyses in zebrafish have identified Notch3 signaling as a key pathway that maintains radial glial quiescence (Alunni et al., 2013). Notch signaling maintains NSC quiescence in constitutive niches (SEZ and SGZ) of the adult mouse as well (Imayoshi et al., 2010), although it is not yet clear which Notch receptor is involved. In the newt, blocking systemic Notch signaling has been shown to lead to an increased number of proliferating pallial radial glial cells (Kirkham et al., 2014). In both mouse and zebrafish, however, Notch1 signaling is necessary for the maintenance of activated (proliferating) NSCs, controlling either cell division or ‘stemness’ (Pierfelice et al., 2011; Alunni et al., 2013). Other molecular components of the NSC quiescence cascade identified in zebrafish include the transcription factors Id1 and Fezf2 (Berberoglu et al., 2014; Rodriguez Viales et al., 2015). Both factors are also expressed in adult mouse NSCs, and were specifically associated with increased quiescence (Nam and Benezra, 2009), although their functional role in quiescence control remains to be shown.

### Recruiting niche glial progenitors for neuronal repair

The birth of new neurons via constitutive neurogenesis is not adequate to replenish the sudden loss of neurons that occurs following injury. Here, something greater is required: the recruitment of endogenous glial progenitors to undergo reactive neurogenesis. The zebrafish adult pallium can undergo reactive neurogenesis remarkably well, replacing lost neurons efficiently in all cases of mechanical injury using stab lesions (Ayari et al., 2010; Kroehne et al., 2011; März et al., 2011; Baumgart et al., 2012; Skaggs et al., 2014). The first response to this type of injury is immune cell activation: the number of microglia and leukocytes in the injured pallial hemisphere increases significantly for several days (Baumgart et al., 2012; Kyritsis et al., 2012). Next, ventricular cells are recruited to proliferate. Conditional Cre/lox lineage tracing in which radial glial cells and their progeny were permanently labeled demonstrated that radial glial cells give rise to neuroblasts that migrate to the site of injury, where they differentiate into long-lasting neurons (Kroehne et al., 2011). A recent lineage-tracing study (Box 1) showed how the division mode of NSCs partially switches upon mechanical lesion such that the proportion of symmetric neurogenic divisions, which is favorable to neuronal repair, increases. This is consistent with what is seen in the mouse SEZ (Ohab et al., 2006). These divisions consumed radial glial cells, generating either one cell maintaining *gfap:gfp* expression soon after division and one non-radial glial cell, or symmetric divisions in which two non-radial glial cells were produced (Barbosa et al., 2015). These studies show how radial glia from the endogenously active pallial NSC zone can be efficiently stimulated and re-routed towards brain repair (Fig. 2), a process that involves several distinct molecular pathways (Table 1).

**Table 1.**
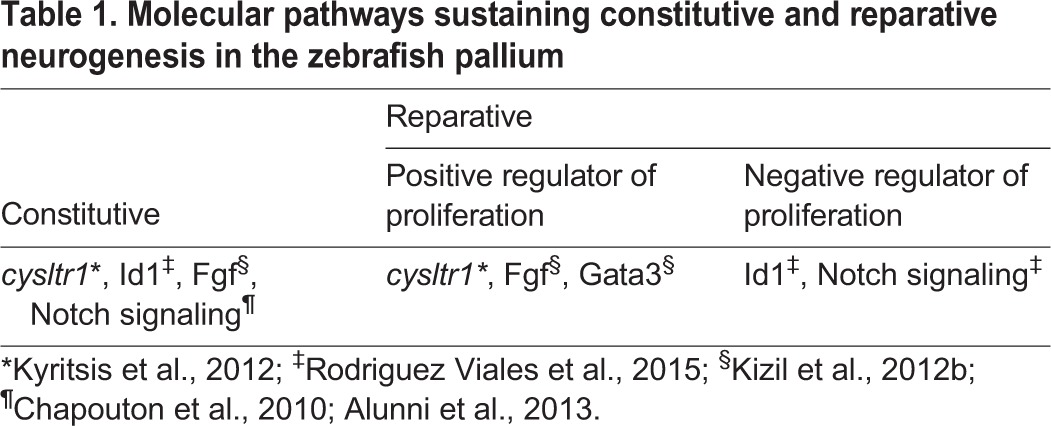
**Molecular pathways sustaining constitutive and reparative neurogenesis in the zebrafish pallium**

To date, recruiting endogenous progenitors is also the most successful strategy to restore neuronal function in rodents. Stroke injury in the rodent striatum leads to increased proliferation in the SEZ, an increased proportion of neurogenic divisions, the redirection of cell migration towards the striatum, and neuronal differentiation into striatal medium spiny neurons (Ohab et al., 2006). Light-induced apoptosis induction in corticospinal projection neurons also triggers the re-routing of SEZ neuroblasts towards the cortex, which is accompanied by some functional regeneration (Chen et al., 2004) (Fig. 2). Finally, ischemia-induced apoptosis of hippocampal CA1 pyramidal neurons stimulates the activation of endogenous SGZ neural progenitors in the dentate gyrus and their subsequent migration into the CA1 layer, leading to functional recovery, albeit incomplete (Nakatomi et al., 2002) (Fig. 2). Although it is clear that recruitment of endogenous neural progenitors can, in some cases, lead to functional recovery, the process remains inefficient, and there is still much to learn about the mechanisms that enhance the mobilization of these cells for repair. How is the migration of these progenitors channeled towards injury sites and how is their fate reoriented towards producing neuronal subtypes that match those of the missing neurons? How do these progenitors overcome anti-neurogenic influences when settling within non-neurogenic areas? The coordination of neurogenesis and angiogenesis appears to be crucial, and some of the factors controlling neuroblast recruitment and migration, such as brain-derived neurotrophic factor (BDNF), have been recently identified (Grade et al., 2013).

### Shared and divergent processes in glial cell-mediated vertebrate neuronal repair

#### A dual role for inflammation

Inflammation is a necessary first response to injury in many tissues, but whether it acts for better or for worse depends very much on the context. In the adult zebrafish pallium, the inflammatory response is key for initiation of specific regeneration programs. Analysis of gene expression via transcriptome screening and *in situ* hybridization before and after mechanical lesion in the zebrafish adult pallium identified candidate genes involved in the regenerative response (Kizil et al., 2012c; Rodriguez Viales et al., 2015). One of these, the transcription factor Gata3, is known to be regulated by active Fgf signaling and inflammation (Kyritsis et al., 2012). Gata3 was found to be specific to the post-traumatic state: its expression was rapidly induced in radial glial cells following lesion, and abrogation of Gata3 activity blocked radial glial activation and decreased regenerative neurogenesis (Kizil et al., 2012c). As Gata3 may be involved in regeneration in other tissues, it will be particularly interesting to determine the downstream mediators and partners of Gata3 in the regenerating brain (Kizil et al., 2012c). The chemokine receptor Cxcr5, expressed by radial glial and periventricular cells, is also involved in the regenerative neurogenesis response, indicating a role for chemokine signaling in this process (Kizil et al., 2012a).

In striking contrast to zebrafish, the neuroinflammatory reaction that follows traumatic brain injury in mouse and human promotes the formation of a deleterious glial scar, and is a direct negative regulator of neurogenesis (for recent reviews, see Kyritsis et al., 2014; Kizil et al., 2015). The fact that neuroinflammation can elicit seemingly opposite outcomes in different vertebrate species and/or lesion modes is particularly interesting, and it will be important to understand how this occurs. Upon lesion, reactive macroglial cells, notably astrocytes, which can be directly recruited or generated from ependymal cells, upregulate the expression of intermediate filament proteins such as GFAP and vimentin, fill up some of the wound with their hypertrophic processes and deposit extracellular matrix and proteoglycans, which impede regeneration (Burda et al., 2016). Astrocytes are not found in the zebrafish brain, but large injuries, in particular those performed through the skull and allowing invasion of the parenchyme by the cerebrospinal fluid, also trigger Gfap upregulation in zebrafish pallial radial glia. Interestingly, however, this does not appear to be followed by the formation of a scar (März et al., 2011; Kishimoto et al., 2012). A number of studies have showed that neuroinflammation and, more specifically, microglial activation in mouse decreases the proliferation of reactive neural progenitors and impairs neuronal differentiation, survival and integration (Iosif et al., 2008; Jakubs et al., 2008). However, more recent reports challenge this view, identifying an early inflammatory response mediated by chemokine signaling that is beneficial for neurogenesis (reviewed by Jaerve and Müller, 2012). Overall, lesion-induced inflammation appears to be long lasting in mammals, with an acute phase followed by a chronic stage, whereas it resolves relatively quickly in fish. This difference might explain why inflammation in zebrafish has a positive effect on endogenous neural progenitors, whereas it has a negative impact in mammals. There is no mechanistic understanding of this difference at present, but an in-depth analysis of the immune cell types and molecular mediators involved, as well as their effects on the variety of macroglial cells of the central nervous system (Anderson et al., 2014) will be an important step towards this goal.

#### The many faces of the Notch signaling pathway

The role of Notch signaling in constitutively active germinal niches under homeostatic conditions is complex both in zebrafish and in mouse, and must be considered separately for each of the different Notch receptors. In the intact adult zebrafish pallial germinal zone, Notch1 is expressed in activated radial glia and maintains their stemness (Alunni et al., 2013). Likewise, after lesion, Notch1 expression is increased in actively proliferating subpallial neural progenitors and is necessary for their maintenance (Kishimoto et al., 2012). *notch3* and its potential downstream target *her4* are also upregulated upon lesion in the zebrafish pallium; however, this is somewhat counter-intuitive, as Notch3 promotes quiescence under normal homeostatic conditions (Alunni et al., 2013). *id1* is also upregulated after injury, but its functional abrogation enhances the proliferative response of radial glia to the lesion (Rodriguez Viales et al., 2015). These two examples suggest that, upon mechanical injury, genetic pathways that counteract reparative neurogenesis by enforcing progenitor cell quiescence are induced. One possible interpretation of this is that the reaction is necessary to limit radial glial cell recruitment for repair, thus avoiding exhaustion of the NSC pool. By inflicting repetitive lesions to the zebrafish pallium it might be possible to test this hypothesis, which would help to answer some very important questions. First, the increased expression of reactive *notch3* and *id1* apparently takes place in all reactive radial glial cells upon lesion, so why do only some radial glial cells bypass this in order to divide? More generally, does the mosaic efficiency of this process reflect some intrinsic radial glial heterogeneity, or is there some mechanism for control at the population level? Second, what are the signals responsible for setting up this anti-reparative response, and could they play some role to limit NSC recruitment in mammals? Interestingly, the upregulation of *id1* expression in the injured zebrafish pallium does not rely on inflammatory signals (Rodriguez Viales et al., 2015) and its regulators remain to be discovered.

The role of Notch in the newt forebrain upon lesion has been addressed by systemic blockade with the γ-secretase inhibitor DAPT (Kirkham et al., 2014). In view of the distinct roles played by Notch1 and Notch3, the results obtained in this species remain difficult to interpret. DAPT lowers the proliferative reaction of quiescent radial glial cells upon lesion, but this might reflect the concomitant block of Notch3 and Notch1, resulting in a loss of radial glial stemness upon activation (Kirkham et al., 2014). The same interpretation may account for a similar observation in the zebrafish subpallium (Kishimoto et al., 2012). Furthermore, the lesion paradigm used in the newt was based on deletion of choline acetyltransferase (ChAT)-expressing neurons located in the pre-optic area of the forebrain (Kirkham et al., 2014). Thus, because radial glia are probably recruited close to the lesion and not in the pallium proper, it may also be that Notch signaling plays a different role in this lesional context and/or location. It will be very interesting to decipher the basis for the apparent discrepancy between the net effects of Notch signaling in these different non-mammalian contexts. A role for Notch in the mobilization of constitutively active NSCs during repair in mammals remains to be studied.

#### Nerve-derived cues stimulate endogenous pallial neurogenic niches

Regeneration assays in the axolotl brain usually involve drastic lesions that ablate large portions of the pallium. These lesions regenerate within 12-15 weeks, but only when the olfactory nerve is preserved, or after it has regrown to re-establish contact with telencephalic tissue (Maden et al., 2013). When the olfactory nerve does not maintain or make contact with telencephalic tissue, only wound healing takes place. These results suggest that olfactory nerve-derived cues produced at a short range promote the recruitment of neural progenitor cells to mediate telencephalic tissue regeneration. As to exactly which cells are recruited and the precise nature of the stimulating cues, these remain important points to be addressed. A number of studies have revealed the importance of nerve-derived cues, such as neurotrophins and in particular BDNF, on the proliferation of neural progenitors under physiological conditions in rodents (reviewed by Berg et al., 2013). The dependency of axolotl pallial regeneration on a cue derived specifically from the olfactory nerve and not simply released by other local neurons might allow the identification of a novel nerve-derived factor, or a specific dosage or location of this factor, that could stimulate regeneration under reparative conditions in mammals as well.

### A novel regenerative perspective: a possible role for constitutive neuro-epithelial niches in neuronal repair

Non-glial cells with neuroepithelial characteristics can be found in some regions of the adult non-mammalian central nervous system and also serve as adult NSC-like progenitors under physiological conditions (for a review, see Than-Trong and Bally-Cuif, 2015). In *Xenopus* as well as adult zebrafish and medaka, these progenitors reside in the ciliary marginal zone (CMZ), as well as at the margin of the optic tectum of the adult zebrafish and medaka brain (Alunni et al., 2010; Ito et al., 2010) and the lateral edge of the zebrafish pallium (Dirian et al., 2014). Although the properties of these neuroepithelial cells have not been extensively tested, they do appear to rely on different pathways of maintenance compared with radial glial cells (Dirian et al., 2014), and they have been shown to be involved in regeneration. For example, the CMZ appears as the main source for retinal regeneration in *Xenopus tropicalis* (Miyake and Araki, 2014) (Fig. 2). At present, evidence to support the maintenance and functional relevance of such cells in the adult mammalian central nervous system is scattered, but suggestive: cells expressing progenitor markers can be induced in human retinal explants in a location homologous to the CMZ (Bhatia et al., 2009); a Notch1-independent neuroepithelial territory was observed at the posterior edge of the mouse optic tectum (Lutolf et al., 2002); and, finally, neuroepithelial progenitors able to generate a subset of adult SGZ NSCs at late embryonic stages were recently identified in the mouse hippocampus, in a location possibly homologous to the zebrafish pallial neuroepithelial pool (Li et al., 2013). Future studies should address and compare the highly important issue of regeneration from adult neuroepithelial pools in the brain and eye of both mammalian and non-mammalian species.

## Recruiting latent progenitors for repair

Some regenerative responses in non-mammalian species do not involve constitutively active neurogenic niches but rather appear to recruit latent progenitors (Fig. 1, blue). The best-studied example of this is in the retina, where a specialized population of glial cells, the Müller glia, act as a major neurogenic source upon lesion despite the fact that they are very seldom neurogenic under homeostatic conditions (Fausett and Goldman, 2006; Raymond et al., 2006; Bernardos et al., 2007; Fimbel et al., 2007). In the newt, full retinal regeneration following ablation is achieved via the de-differentiation of a different cell population, the retinal pigmented epithelium (Stone, 1950; Hasegawa, 1965). Two other relevant cases, the spinal cord of the zebrafish and the midbrain in the newt, further exemplify the reparative recruitment of latent cells, in this case, the ependymoglial cells that line the brain ventricle or central canal (Reimer et al., 2008; Berg et al., 2010). Comparing these examples and the molecular pathways that regulate them (Table 2) may provide useful insight into possible mechanisms to stimulate dormant progenitors for neuronal repair in mammals. Interestingly, recent data in rodents demonstrated the plasticity of endogenously non-neurogenic cells, such as ependymal cells or parenchymal astrocytes, in response to lesion in the brain or spinal cord (Buffo et al., 2008; Carlen et al., 2009; Barnabe-Heider et al., 2010; Sirko et al., 2013; Magnusson et al., 2014).

**Table 2.**
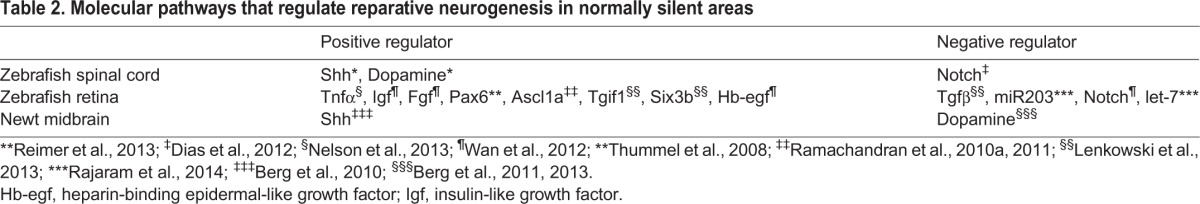
**Molecular pathways that regulate reparative neurogenesis in normally silent areas**

### Reactivation of latent progenitors for neuronal repair in the retina

**Fig. 3.**
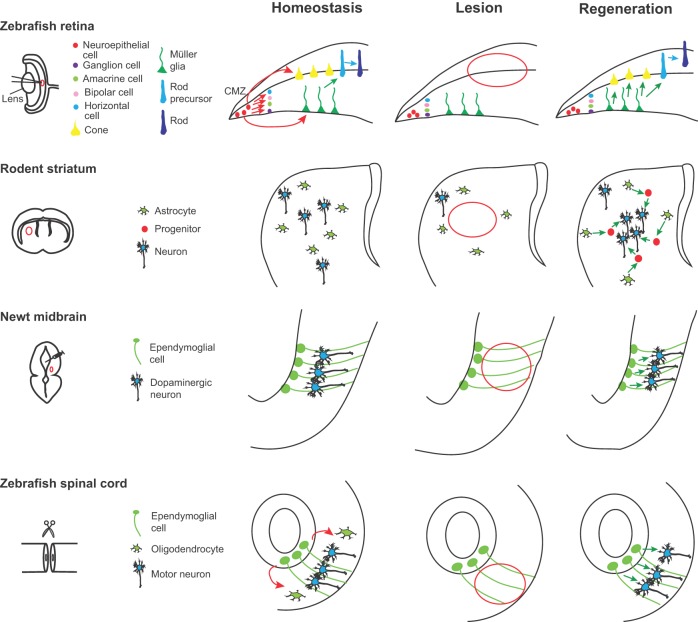
**Neuronal repair from latent progenitors.** In the zebrafish retina, new retinal neurons are generated sequentially from retinal stem cells located in the ciliary marginal zone (CMZ). Under homeostatic conditions, the Müller glia cells (MGs) generate only rod precursors, which give rise to rod photoreceptors (left panel, green and blue arrows). Following lesion (center panel, red outline), MGs re-enter the cell cycle and divide once asymmetrically to generate neurogenic clusters that go on to produce all missing neurons (right panel, green arrows). In the rodent striatum, the astrocytes are not neurogenic (left panel). After a stroke (center panel, red outline), some striatal astrocytes generate neuroblasts that give rise to a limited number of new neurons (right panel, green arrows). In the newt midbrain under homeostatic conditions, ependymoglial cells are quiescent (left panel). A selective neurotoxin administered intraventricularly selectively eliminates midbrain dopaminergic neurons (center panel, red outline), inducing the proliferation of the ependymoglial cells, which generate new dopaminergic neurons (right panel, green arrows). Ependymoglial cells in zebrafish spinal cord are self-renewing and give rise to oligodendrocytes (left panel, red arrows). After a lesion (center panel, red outline), these cells divide, migrate and produce new motor neurons (right panel, green arrows).

The retina grows during the entire life of teleost fish by addition of new neurons, originating from retinal stem cells of the CMZ (Fig. 3). In addition, Müller glial cells, distributed all over the differentiated retina, divide very infrequently in the adult to produce new rod photoreceptors (Raymond et al., 2006) (Fig. 3). A key signaling pathway imposing Müller glia quiescence under physiological conditions is Notch (Conner et al., 2014). In lesional contexts, Müller glia are the source of retinal progenitor cells driving regeneration of retinal neurons (Bernardos and Raymond, 2006; Fausett and Goldman, 2006; Bernardos et al., 2007; Fimbel et al., 2007; Ramachandran et al., 2010b): they re-enter the cell cycle and divide asymmetrically to generate neurogenic clusters that then give rise to all the missing neurons (Nagashima et al., 2013). In this way, stimulated Müller glia cells display a much greater lineage repertoire than under homeostatic conditions, as they must produce an array of neuronal lineages that they do not usually make. Inhibition of the Müller glia cells during regeneration results in regenerative failure (Thummel et al., 2008) (Fig. 3).

The molecular cascades that drive Müller glia-mediated repair in zebrafish are certainly the most studied in the context of stimulating latent progenitors, and so deserve some detailed discussion in addition to recent reviews (Goldman, 2014; Lenkowski and Raymond, 2014). Several signaling pathways activated upon lesion stimulate Müller glia re-entry into the neural progenitor mode, including Tumor necrosis factor (Tnfα), which is produced by dying retinal neurons and further stimulates the production of other growth factors and cytokines at the injury site (Nelson et al., 2013). These factors converge onto the activation of the mitogen-activated protein kinase (MAPK) and phosphoinositide 3-kinase (PI3K) pathways, followed by activation of β-Catenin and Stat3. In parallel, two co-repressors, Tgif1 and Six3b, which are rapidly upregulated prior to the first Müller glia division, repress Tgfβ signaling in Müller glia through Smad2/3 to permit the proliferative response and limit gliosis, the non-specific pathological remodeling of glia cells in response to damage (Lenkowski et al., 2013; Lenkowski and Raymond, 2014). In addition, upregulation of another transcription factor, Ascl1, is a key event in Müller glia activation. One important downstream effector of Ascl1 is lin28, which contributes to the Müller glia response through a negative-feedback loop: it decreases let-7 miRNA levels, relieving repression of regeneration-associated mRNAs, including *klf4*, *oct4* (*pou5f3* – Zebrafish Information Network) and *cmyc* (*myca* – Zebrafish Information Network), which are components of well-known reprogramming cocktails (Ramachandran et al., 2010b). The Ascl1/lin28 pathway also results in induction of expression of Pax6, required upon repair for increased progenitor cell proliferation (Thummel et al., 2008; Rajaram et al., 2014). Finally, Ascl1 also induces expression of the transcription factor Insm1, which helps promote Müller glia re-entry into cycle (Ramachandran et al., 2011).

Apart from fish and urodeles, functional repair of damaged retinas has seldom been demonstrated in vertebrates. Rodent Müller glia proliferate and express genes associated with retinal stem cells in response to injury, but generally do not themselves function as retinal progenitors *in vivo* (Jadhav et al., 2009). Rather, they have been shown to undergo gliosis (Dyer and Cepko, 2000; Bringmann et al., 2009). Following retinal injury, mouse Müller glia do not express Ascl1; however, when forced to overexpress Ascl1 in culture they re-express progenitor genes, including *Insm1*, and re-enter the cell cycle to generate amplifying neuronal progenitors and neurons (Pollak et al., 2013). A recent report further demonstrates that forced expression of Ascl1 in Müller glia upon lesion in young mice is sufficient to drive them towards efficient neurogenesis (Ueki et al., 2015). Thus, a major difference in the regulation of Ascl1 expression in Müller glia upon lesion might underlie the difference in success of retinal regeneration between mouse and zebrafish.

Several important issues are raised by these data. First is the question of whether the lesion-induced molecular cascades in zebrafish Müller glia are truly lesion specific, or whether certain genes are expressed at low levels under basal conditions. Some Ascl1-induced pluripotency factors are endogenously expressed at low levels in zebrafish Müller glia (Ramachandran et al., 2012) and their promoters are hypomethylated even in the absence of lesion (Powell et al., 2013), whereas the promoters of progenitor genes are epigenetically silenced in mouse Müller glia (Pollak et al., 2013). A detailed comparison of the basal status of zebrafish versus mammalian Müller glia, or mammalian astrocytes, may bring to light some molecular targets for the recruitment of latent mammalian progenitors. Another issue is understanding how reactive zebrafish Müller glia are able to adjust their response to regenerate the appropriate number and subtypes of neurons. A possible hypothesis here is that, to permit proper neuronal patterning following reprogramming, Ascl1 induction must be transient, which may be permitted by negative-feedback loops involving Insm1 and Ascl1 (Ramachandran et al., 2012). If this is the case, then it might be possible to reconstruct such a molecular loop in mammalian Müller cells in order to promote retinal regeneration over gliosis. Another issue that must be addressed is the role of Tnfα in promoting progenitor survival (Conner et al., 2014), and the additional pathways and components that are involved, independent of Notch inhibition. Finally, an important aspect of the reparative reaction in the retina is also to limit it. This is crucial to prevent stem cell exhaustion, overgrowth and the development of tumors, and/or the establishment of a long-lasting gliosis reaction. In zebrafish, a second peak of Notch-dependent *insm1* expression contributes to stopping the proliferative reaction by promoting differentiation (Ramachandran et al., 2012). In both reactive and homeostatic Müller glia, Notch itself also limits proliferation through the inhibition of Ascl1 and Stat3 expression (Conner et al., 2014). Whether and how this reaction is conserved across species remains unclear, as Notch has been shown to activate Müller glia in rodents and chicken (Hayes et al., 2007; Del Debbio et al., 2010).

### Reactivation of latent progenitors for neuronal repair in the brain

The discovery that latent but reactivatable progenitor cells also exist within virtually all brain subdivisions in rodents further generated great hopes to mobilize these endogenous cellular sources for repair (reviewed by Robel et al., 2011), although it must be noted that regenerative neurogenesis in rodents remains far less efficient than in non-mammalian species. Among the major candidate cell types for repair are ependymal cells and astrocytes, both of which react to injury by re-expressing progenitor markers and by proliferating *in vivo*, and which exhibit multipotency at least *in vitro* (Buffo et al., 2008; Carlen et al., 2009; Barnabe-Heider et al., 2010; Sirko et al., 2013; Magnusson et al., 2014). Upon spinal incision in the adult mouse, ependymal cells located in the vicinity of the lesion site activate and re-orient their fate towards generating new ependymal cells and astrocytes, as well as a low number of oligodendrocytes (Barnabe-Heider et al., 2010). In the forebrain, ependymal cells lining the lateral ventricle also reactivate after stroke and re-orient their fate to generate astrocytes and neuroblasts in the lateral wall (Carlen et al., 2009). Both examples demonstrate the remarkable activation potential and fate plasticity of ependymal cells *in vivo* and identify them as interesting targets for manipulation of cell fate towards making neurons. In a similar way, astrocytes located in the cortical and striatal regions of the brain as well as in the spinal cord parenchyma also exhibit an impressive plasticity, as the fate of these cells can be re-oriented towards neurogenesis upon forced expression of some transcription factors, such as Sox2 (Niu et al., 2013), or a combination of Ascl1, Brn2 (Pou3f2 – Mouse Genome Informatics) and Myt1l (Torper et al., 2013). This plasticity can also be seen in some lesional contexts, in particular middle cerebral artery occlusion-induced stroke or mechanical lesions (Buffo et al., 2008; Sirko et al., 2013; Magnusson et al., 2014), although the local origin of these neurogenesis-competent astrocytes has recently been questioned (Faiz et al., 2015). Finally, the neurogenic potential of other macroglial cell types, such as a subset of parenchymal glial cells expressing the NG2 (chondroitin sulfate proteoglycan 4) antigen, known as NG2 glia, has recently been identified. NG2 glia are the main proliferating macroglial cell type in the intact adult brain, and are normally largely fated to the generation of NG2 glia and oligodendrocytes, as well as some astrocytes in restricted brain areas (Guo et al., 2014; reviewed by Dimou and Gotz, 2014). Importantly, the translational relevance of these mouse studies is reinforced by the fact that postmortem material from patients who suffered stroke, hemorrhage or some neurodegenerative diseases can display signs of reactive proliferation, re-expression of neural progenitor markers, and neurogenesis (reviewed by Robel et al., 2011), although the process is probably abortive (Huttner et al., 2014).

These studies demonstrate some heterogeneity within macroglial cells in response to a reactive context, and point to the different propensity for reactivation depending on the lesional context (Sirko et al., 2013; Guo et al., 2014). In spite of this, and as seen in the retina, a common denominator of successful neuroblast formation is Ascl1 (Magnusson et al., 2014). Furthermore, very recent data demonstrated that Ascl1 induces Insm1 expression during the forced conversion of adult mouse cortical astrocytes, human astrocytes or mouse fibroblasts into GABAergic neurons, and that Insm1 is a necessary component of this reprogramming process (Masserdotti et al., 2015). In fact, in combination with NeuroD4, Insm1 is also sufficient for reprogramming. These results are in line with the early role of Insm1 in reactive Müller glia in zebrafish, and further support the notion that activation of the nodal pair Ascl1/Insm1 is a fundamental reprogramming event upon lesion, conserved across species. Additional data from these studies highlight the importance of downregulating Notch1 signaling to permit reactivation and/or proliferation and/or acquisition of a neuroblast fate in both ependymal cells of the lateral wall and striatal astrocytes (Magnusson et al., 2014) (Fig. 3). Understanding the different possible functions of Notch in these contexts will be an important task for future studies.

Regeneration from latent progenitors in the adult brain in non-mammals has not been extensively studied, except for the loss and subsequent regeneration of dopaminergic and cholinergic neurons in the newt midbrain. Stereotaxic injection of the neurotoxin 6-hydroxydopamine (6-OHDA) into the brain ventricle, which eliminates midbrain dopaminergic neurons, was followed by complete regeneration in adult newts (Fig. 3) (Berg et al., 2010). The source of the new neurons was shown to be ependymoglial cells, which are present along the midbrain ventricle, and have radial glia morphology and express GFAP. Constitutive neurogenic niches in the adult newt are restricted to the forebrain, and thus ependymoglial cells are normally quiescent. However, both sham and 6-OHDA injections triggered the re-entry of midbrain ependymoglial into the cell cycle, and in the latter case, proliferation was sustained and led to neurogenesis and functional repair (Parish et al., 2007). In a separate study, ablation of cholinergic neurons upon injection of ethylcholine aziridinium resulted in regeneration of the neurons after 7 weeks, again following a phase of induced ependymoglial proliferation in the midbrain ventricles (Berg et al., 2011). In this latter study, the authors demonstrated the importance of dopamine in the regenerative response. Following the destruction of dopaminergic neurons in the newt midbrain, injection of the dopamine mimic L-dopa blocked the proliferative response of ependymoglial cells (Berg et al., 2011). Interestingly, this did not affect the ependymoglial cell response to the destruction of cholinergic neurons, and appears to have been exerted directly on midbrain ependymoglial cells, probably via their expression of the dopamine receptor D2. The authors proposed that the role of dopamine was to act within a negative-feedback mechanism to match the production of dopaminergic neurons with the size of the existing midbrain dopaminergic pool (Berg et al., 2011, 2013). Importantly, the effect of dopamine is not limited to the regenerative context, as under physiological conditions dopamine acts to promote ependymal cell quiescence (Berg et al., 2011), and in mammals, dopamine promotes neurogenesis in active neurogenic zones (Kippin et al., 2005; O'Keeffe et al., 2009). As in the newt, the destruction of midbrain dopaminergic neurons by 6-OHDA in rats also leads to the activation of dormant midbrain progenitors, but these cells fail to regenerate dopaminergic neurons *in situ* (Lie et al., 2002).

Another important player in this context is Sonic Hedgehog (Shh). Shh expression was also induced in ependymoglial cells at the midbrain ventricle upon ablation of dopaminergic neurons by 6-OHDA in the newt. Furthermore, cyclopamine inhibition of Shh signaling reduced dopaminergic neuron regeneration, possibly through targeting progenitor specification or differentiation (Berg et al., 2010). Shh signaling plays multiple context-dependent roles during neurogenesis in vertebrate embryos and adults, impacting patterning, proliferation, cell division mode, cell cycle exit and progenitor migration (Ferent and Traiffort, 2015). In the context of rodent brain regeneration, it has so far only been implicated as a proliferation-inducing factor in mouse cortical astrocytes (Sirko et al., 2013).

### Recruiting latent progenitors for repair in the spinal cord

Under normal conditions, the adult zebrafish spinal cord shows very little, if any, cell proliferation and neurogenesis, which is consistent with what is observed in the mammalian spinal cord (Yamamoto et al., 2001b). In contrast to mammals, however, neurons are generated in high numbers in the zebrafish spinal cord after a lesion (Fig. 3). A possible source of these cells is the ependymoglial cell population. These cells line the central canal in the adult zebrafish spinal cord and have a radial glial morphology, expressing Blbp (Fabp7a – Zebrafish Information Network) (dorsally) and/or the transcription factor Olig2 (in a more ventral domain) under normal physiological conditions. These ependymoglial cells normally divide slowly and asymmetrically to self-renew and produce oligodendrocytes (Park et al., 2007), but following a lesion, Olig2-positive ependymoglial cells re-enter the cell cycle, migrate, and differentiate into mature motor neurons (Reimer et al., 2008). Lineage-tracing studies performed during spinal cord regeneration in axolotl led to similar conclusions (McHedlishvili et al., 2007).

Recent studies have uncovered some of the key molecular players involved in activation of ependymoglial cells and subsequent spinal cord regeneration. One of the earliest factors expressed in activated zebrafish ependymoglial cells following injury is Sox2, which is required for re-initiation of proliferation (Ogai et al., 2014). A similar function for Sox2 was also demonstrated in the axolotl (Fei et al., 2014). Sox11b is also upregulated in zebrafish ependymoglial cells lining the central canal, and is necessary for their reactive proliferation and for locomotor recovery after spinal cord injury (Guo et al., 2011). Notch pathway genes are also upregulated after zebrafish spinal lesion in ependymoglial cells, but in this case they function to reduce progenitor cell proliferation and motor neuron generation. This is reminiscent of its reaction-antagonizing function following injury in the pallium and retina (Conner et al., 2014; Rodriguez Viales et al., 2015). Importantly, however, in the spinal cord, Notch manipulations have no effect on ependymoglial cells under normal physiological conditions (Dias et al., 2012), suggesting that quiescence is maintained in these cells by a mechanism that differs from other central nervous system areas and/or cell types. As previously discussed, spinal cord lesions in the adult rodent are followed by the reactivation of ependymoglial cells lining the central canal, which go on to generate astrocytes and oligodendrocytes but no neurons (Barnabe-Heider et al., 2010). Previous studies demonstrated an upregulation of Notch signaling in these ependymoglial cells post-lesion, and a role for Notch in inhibiting the acquisition of a neuronal fate (Yamamoto et al., 2001a). Whether Notch also plays a role in limiting ependymoglial cell activation in this system remains to be tested.

As previously demonstrated in the brain, another important player of spinal cord regeneration is dopamine. During regeneration in zebrafish, dopaminergic axons that descend from the brain were shown to undergo sprouting rostral to the lesion site (Reimer et al., 2013). In line with this, *in situ* hybridization indicated lesion-induced upregulation of the dopamine D4a receptor gene in the ependymal progenitor zone of the spinal cord segment located rostral to the lesion. Injection of the dopamine agonist NPA to mimic dopaminergic innervation posterior to the lesion increased motor neuron regeneration. The results from this study highlight a proliferation-promoting and/or neurogenic role for dopamine on quiescent ependymoglial cells during the spinal regeneration process. This action is clearly distinct from the effect of dopamine in the regenerating newt midbrain, and it will be very important to understand how dopamine can exert such disparate effects on similar cell types in the spinal cord and midbrain, and how specificity is achieved to control neuronal subtype generation. In the spinal cord, dopamine acts at least in part through activating the Hedgehog pathway (Reimer et al., 2013). The endogenous, albeit weak, expression of Shh and dorsoventral patterning markers in ependymoglial cells of the zebrafish adult spinal cord suggests that a latent embryonic positional information program persists in these cells. A similar conclusion was obtained in the axolotl and the newt (Schnapp et al., 2005). In addition, Shh expression is strongly increased in the ventral ependymoglial cells from which motor neurons regenerate, located in the vicinity of a spinal cord lesion in the adult zebrafish and adjacent to a more dorsally induced domain of strong Pax6 expression. Unlike in the midbrain, however, where blocking Shh signaling *in vivo* did not affect the lesion-induced proliferation of ependymoglial progenitors, it does so in the spinal cord, and further impairs motor neuron regeneration. As such, Shh is clearly an important factor for promoting the activity of ependymoglial cells as motor neuron progenitors (Reimer et al., 2009). Whether the primary function of Shh in this context is repatterning, induction of proliferation and/or reprogramming, remains to be assessed.

Many other cellular events contribute to regeneration after spinal cord injury including inflammation, cell death, proliferative response, neurogenesis and axonal regrowth (Hui et al., 2014). Given the capacity for rodent spinal ependymoglial cells to generate neurons *in vitro* but not *in vivo* (Barnabe-Heider et al., 2010), it is interesting to consider not only cell-intrinsic factors but also environmental cues that may bias progenitor fate in zebrafish. Several studies showed that during the spinal regeneration process, inhibitory components, such as chondroitin sulfate proteoglycans (Fawcett et al., 2012) and myelin-associated inhibitory molecules normally present in mammals, are absent in zebrafish (Becker and Becker, 2014). This may contribute to generating an environment that is permissive for neurogenesis and/or neuronal survival in zebrafish. In addition, pericytes are prominently involved in scar formation in the injured rodent spinal cord, a process that impairs regeneration (reviewed by Sabelstrom et al., 2013). The nature and fate of pericytes in the zebrafish spinal cord upon lesion remains to be studied.

## Conclusions

The study of non-mammalian models, in which the repertoire of NSCs and latent progenitors is largely similar to that of rodents, has yielded important molecular insights into the mechanisms of neuronal repair across species. The roles of Ascl1, Shh and Notch signaling, initially studied for their importance in progenitor recruitment for repair in zebrafish or newts, appear to be conserved in some neural regenerative contexts in rodents. In addition, studies conducted in zebrafish suggest a synergy between constitutive neurogenic pathways and regeneration-specific molecular events during repair. For example, the early inflammation reaction in response to a mechanical lesion or physically induced neuronal death is an important upstream component of the regeneration-specific cascade and includes the induction of *gata3* expression in constitutively active pallial NSCs, or the partial reprogramming towards a progenitor state of latent retinal Müller glia. Finally, the existence of feedback loops that partially limit endogenous progenitor cell recruitment to select subpopulations in virtually all regions studied in non-mammalian models sheds light on the issue of progenitor heterogeneity. Understanding the cell-intrinsic and environmental components that drive the heterogeneity and the spatial organization of the reactive response is likely to involve the dynamic analysis of different progenitor pools within their three-dimensional niche both under native and reparative conditions, a feat that is now technically feasible in zebrafish (Box 1).

Overall, one key message of this Review is that the regulation of the processes essential to adult neural progenitor function, such as the molecular cascades that control their quiescence and activation, their proliferation and division modes, and, most likely, the early lineage decisions of radial astroglial cells, are highly conserved between vertebrate species, under both physiological and reparative conditions. Despite these commonalities, it is important to remember that the translation of these studies into humans is a distinct step, whether starting from non-mammals or from rodents. Indeed, specific features that distinguish primate NSCs from rodent NSCs have recently been highlighted and one needs to avoid generalizations between taxons. For example, the activity of a SEZ-like domain in humans is heavily debated (reviewed by Jessberger and Gage, 2014), and the astrocytic repertoire of rodents is much less complex than in humans (Colombo et al., 1997; Oberheim et al., 2009; Sosunov et al., 2014). Likewise, the development of the primate cortex, and perhaps that of the human cortex as well, involves progenitor subtypes and cellular processes that have not yet been identified in rodents. These differences between the regulation of neurogenesis in rodents and humans highlight the importance of studying neurogenesis in multiple different model systems. Only by doing so will it be possible to compile a comprehensive set of molecular targets for human regenerative neurogenesis, including those present in non-mammals but not present in rodents. That said, important factors such as the distance of the progenitors from the lesion and the age of the animal compared with critical cell plasticity periods have a different order of magnitude in man compared with any non-primate model. These non-conserved structural and temporal features are clearly a drawback of using animal models, including non-mammals, and should be kept in mind.

Despite commonalities among species with regard to core neural progenitor activity and function, later reparative events strongly differ in their outcome across species. For example, the inflammation reaction in zebrafish generally subsides before the formation of a glial scar, a reactive process that occurs in rodents. Additionally and in contrast to rodents, all the territories of the adult zebrafish central nervous system, even those that are not constitutively neurogenic, seem permissive to the late steps of reparative neurogenesis, including the differentiation and integration of long-lived neurons. Currently, these differences in reparative potential between species are not well understood, and studying non-mammalian models in a comparative manner may bring important insight into this area. A major challenge for the future is to dissect the cellular and molecular mechanisms that regulate the inflammatory reaction in zebrafish and to understand the reasons for its early termination. Because astrogliosis in mammals can be beneficial to limit inflammation and protect neuronal survival (Sabelstrom et al., 2013), an issue when using reactive astrocytes or ependymal cells for neuronal repair will thus be to preserve this protective response and balance gliosis and fate conversion towards neurogenesis (Robel et al., 2011). Finally, another key issue remains that of reparative neurogenesis under neurodegenerative conditions. Although this is the focus of intense investigation in rodents, it has remained to date a relatively unexplored area in non-mammalian models. Outside the 6-OHDA newt model discussed above, neurodegeneration models have been generated in zebrafish but their analysis remained limited to early stages – embryonic, larval, or early juvenile. The extent to which such conditional models in adults would trigger regeneration, the extent of the response, and how it would compare with other lesional contexts and within the rodent/mammalian context are all very important further directions.
